# A scaling law for distinct electrocaloric cooling performance in low-dimensional organic, relaxor and anti-ferroelectrics

**DOI:** 10.1038/s41598-017-11633-y

**Published:** 2017-09-11

**Authors:** Yuping Shi, Limin Huang, Ai Kah Soh, George J. Weng, Shuangyi Liu, Simon A. T. Redfern

**Affiliations:** 1 Department of Mechanical and Aerospace Engineering, Hong Kong University of Science and Technology, Clear Water Bay, Kowloon, Hong Kong; 2 Department of Chemistry, South University of Science and Technology of China, Shenzhen, 518055 China; 3 School of Engineering, Monash University Malaysia, Bandar Sunway, 46150 Malaysia; 4 Department of Mechanical and Aerospace Engineering, Rutgers University, New Brunswick, New Jersey, 08903 USA; 5 Chongqing Institute of Green & Intelligent Technology, Chinese Academy of Sciences, Chongqing, 400714 China; 6 Center for High Pressure Science and Technology Advanced Research, Shanghai, 201203 China; 7 Department of Earth Sciences, University of Cambridge, Downing Street, Cambridge, CB2 3EQ UK

## Abstract

Electrocaloric (EC) materials show promise in eco-friendly solid-state refrigeration and integrable on-chip thermal management. While direct measurement of EC thin-films still remains challenging, a generic theoretical framework for quantifying the cooling properties of rich EC materials including normal-, relaxor-, organic- and anti-ferroelectrics is imperative for exploiting new flexible and room-temperature cooling alternatives. Here, we present a versatile theory that combines Master equation with Maxwell relations and analytically relates the macroscopic cooling responses in EC materials with the intrinsic diffuseness of phase transitions and correlation characteristics. Under increased electric fields, both EC entropy and adiabatic temperature changes increase quadratically initially, followed by further linear growth and eventual gradual saturation. The upper bound of entropy change (∆S_max_) is limited by distinct correlation volumes (V_*cr*_) and transition diffuseness. The linearity between V_*cr*_ and the transition diffuseness is emphasized, while ∆S_max_ = 300 kJ/(K.m^3^) is obtained for Pb_0.8_Ba_0.2_ZrO_3_. The ∆S_max_ in antiferroelectric Pb_0.95_Zr_0.05_TiO_3_, Pb_0.8_Ba_0.2_ZrO_3_ and polymeric ferroelectrics scales proportionally with V_*cr*_^−2.2^, owing to the one-dimensional structural constraint on lattice-scale depolarization dynamics; whereas ∆S_max_ in relaxor and normal ferroelectrics scales as ∆S_max_ ~ V_*cr*_^−0.37^, which tallies with a dipolar interaction exponent of 2/3 in EC materials and the well-proven fractional dimensionality of 2.5 for ferroelectric domain walls.

## Introduction

The electrocaloric effect, i.e. reversible changes in isothermal entropy or adiabatic temperature in polar materials achieved by application and removal of electric fields, has been extensively investigated in the context of next-generation techniques for more efficient and environmentally-friendly solid-state refrigeration and integrable on-chip coolers^1–4^. In addition to the pioneering discovery of giant EC responses in poly(vinylidene fluoridetrifluoroethylene) P(VDF-TrFE)-based polymer^5^ ferroelectrics (FEs) and PbZr_0.95_Ti_0.05_O_3_ antiferroelectric (AFE) thin-films^6^, recent multiscale calculations^7, 8^ and indirect experiments^9–12^ reliant on measuring the thermal dependence of FE hysteresis loops, have reported comparable EC cooling performances in a rich class of EC materials which also include normal and relaxor FEs as well as in a diverse range of low-dimensional structures such as nanotubes^13^, nanowires^14^ and nanocomposites^15^. Although direct EC tests based on *in-situ* measurement of the temperature changes accompanying adiabatic depolarizations have been implemented in FE films and multilayers (typically with microscale thickness)^16, 17^, direct experiments on solid-state cooling responses in low-dimensional EC structures still remains challenging due to their very small heat capacities, deviations from adiabatic conditions and the insufficient yet ultrafast heat transfer to thermal testing units^4, 18^. When it comes to indirect theoretical approaches for determining EC cooling responses, diverse polynomial data fitting and numerical integration techniques for EC performance calculation, together with the intrinsic non-equilibrium nature and complicated kinetic features of both first- and second-order phase transitions, especially in nanostructured EC materials and devices, often produce remarkable discrepancies or even erroneous predictions^3, 4^. Instead, development of a versatile theoretical framework capable of analytically quantifying the intriguing cooling properties in a broad range of EC materials is imperative and timely for the further enhancement of electro-thermal energy converting strength and in the search for new room-temperature refrigeration and microcooler alternatives.

When subjected to electric field (*E*) de-poling effects and temperature (*T*) variations, EC entropy changes (∆*S*) and adiabatic temperature changes (∆*T*) originate from partial reorientation of localized dipoles in organic FEs and from 1^st^ or 2^nd^ order phase transitions in AFEs, normal and relaxor FEs. Consequently, adiabatic depolarization dynamics in the rich variety of EC materials display varied transition diffusenesses and show a huge span in critical dimensions. The latter can range from lattice-scale dipole-dipole correlations to features associated with long-range FE domain structures. Furthermore, the cooling strength of EC effect (ECE) has distinct figures of merits among comprehensive electrically polarizable EC materials - the ECE in normal FEs becomes dramatically enhanced near any sharp phase transition and is seen predominantly around the Curie temperature (*T*_c_); whereas, diffuse phase transitions in relaxor FEs dominated by the evolution of polar nano regions (PNRs) can show significant EC responses over a much broader temperature range^19, 20^, which often takes place far above *T*_c_ and can substantially expand the scope and potential of the EC effect for solid-state and flexible refrigeration. When it comes to organic FEs, P(VDF-TrFE)-based copolymers usually behave like a normal FE; however, its terpolymers with chlorofluoroethylene (CFE) display diffuse dielectric properties^18^ with respect to both varying *E* and *T*. These observations highlight the importance of considering both the overall phase transition features^21^, as well as microscopic correlation characteristics and the lattice symmetry^22^ of diversified EC materials when characterizing their cooling performances whether for academic or industrial motivations.

## Results

The Master equations are introduced here to describe the reorientation dynamics of localized depolarization, in which microscopic correlation characteristics and the lattice symmetry of EC materials are particularly taken into account. The overall polarization magnitude (*P*) and concomitant configurational entropy are treated as a spatially-averaged reflection throughout a sufficiently large ensemble of microscopic polar elements (MPEs), e.g. PNRs, ferroelectric domains or polarizable chemical chains, which correlate with varied strength through electronic or elastic interactions and over different length scales. In our framework, a mean characteristic volume of V_*MPE*_ is particularly considered for an EC material or structure^19, 20^. When *E* is applied along the polarization direction or the axis of lattice symmetry, a universal activation parameter of both *E* and *T* is generalized as $u(E,T)=E{P}_{\max }{{\rm{V}}}_{MPE}/({\rm{\Omega }}kT)$ where *P*_max_, Ω and *k* denote the maximum attainable polarization in the EC material, a symmetry factor and Boltzmann’s constant, respectively. Subsequently, the solution of the established Master equations (see Methods) gives rise to a generic expression for the spatial (*u*) variation of the magnitude of overall polarization as $P={P}_{\max }\,\tanh (u)$, in which the symmetry factor is found to be 1 for normal and organic FEs and 3 for [111]-type depolarizations. The figure of merits of this symmetric polarization formulation with *E* precludes the unwanted complexity of Joule heating induced by non-linear polarization hysteresis, thus offering an opportunity to improve the understanding of cyclically and directly-measured EC cooling performances.

**Figure 1:**
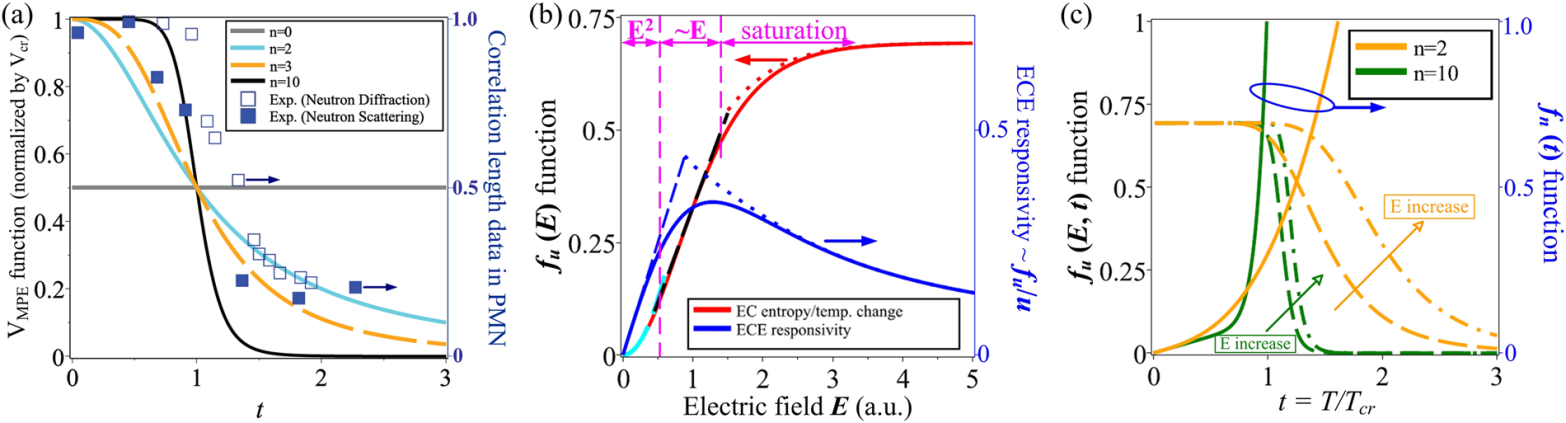
(**a**) Direct comparison of the V_MPE_ function with experimental correlation length data in PMN single crystals measured by neutron elastic diffuse scattering (NEDS) and neutron diffraction (ND) technique. (**b**) Dependence of EC entropy/temperature change (red curve) and ECE responsivity (blue curve) on normalized *E* at a fixed temperature, as given by the *f*_*u*_(*E*, *t*) and *f*_*u*_/*u* function, respectively. (**c**) The *f*_*n*_(*t*) function for ∆T and *f*_*u*_(*E*, *t*) function at *n* = 2 and *n* = 10 vs. *t*, where the hollow arrows point to the increasing direction of *E* and the higher *E* used is ten times the lower *E*. The NEDS and ND correlation length data in (**a**) derived from refs 24 and 25 is normalized by 65 nm and 20 nm, respectively; the T_*cr*_ is chosen as 220 K for the NEDS data and 240 K for the ND data.

In order to embrace the effects of phase transition diffuseness and the Curie temperature on EC cooling strength, the characteristic correlation volume may be related to the thermal diffuseness in the form of ref. 23:

1 $${{\rm{V}}}_{MPE}(T)=\frac{{{\rm{V}}}_{cr}}{1+{(T/{T}_{cr})}^{n}}$$

where *n* is referred as a diffuseness index to distinguish different types of EC phase transitions; V_*cr*_ is the saturated value of V_*MPE*_ at low temperatures; *T*_*cr*_ denotes a critical temperature that can be the Curie temperature for normal FEs or the temperature where the maximum dielectric response occurs in continuously depolarized EC materials. The three-dimensional (3D) plot of equation (1) (Figure SI1) shows that increased diffuseness of V_*MPE*_ dispersion with normalized temperature *t* = *T/T*_*cr*_ accompanies a decreased *n*. This indicates a rather smaller diffuseness index for relaxor FEs than that for sharp transitions in normal FEs. When the EC depolarizations are mediated by indivisible MPEs, say unit lattices or chemical chains, then *n* = 0 is expected to ensure that V_*MPE*_ is constant, which is likely the case for polymeric EC materials. A direct correspondence of our equation (1) to the experimental correlation length data^24, 25^ in typical relaxor Pb(Mg_1/3_Nb_2/3_)O_3_ (PMN) is demonstrated in Fig. 1(a). Since diffuse phase transitions shift the EC ∆*T* and ∆*S* peaks towards higher temperatures above *T*_*c*_, the focal comparison in Fig. 1(a) is made on the *t* > 1 region; the *n* value of well-studied PMN single crystals is shown to lie between 2 to 3, implying a fractional PNR dynamics^26^.

Upon successful establishment of the generic polarization expression with thermally diffused correlation volumes, it is possible for us to elucidate ∆*S* and ∆*T* with the help of Maxwell relations: $dS/dE={(\partial P/\partial T)}_{E}$ and $dT/dE=-\frac{T}{{C}_{{\rm{v}}}}{(\partial P/dT)}_{E}$, where *C*_v_ is the volumetric heat capacity of EC materials. Integrating the two Maxwell relations from zero field to *E* yields

2 $$-{\rm{\Delta }}S=\frac{{\rm{\Omega }}k}{{{\rm{V}}}_{cr}}[1+(n+1){t}^{n}]{f}_{u}(E,t)\,{\rm{and}}\,{\rm{\Delta }}T=\frac{{\rm{\Omega }}k{T}_{cr}}{{{\rm{V}}}_{cr}{C}_{{\rm{v}}}}[t+(n+1){t}^{n+1}]{f}_{u}(E,t)$$

where ${f}_{u}(E,t)=2u-\,\mathrm{ln}(\frac{1+{e}^{2u}}{2})-\frac{2u}{1+{e}^{2u}}$ governs the intrinsic EC responses of electric depolarizations with diffusionless characteristic correlation volumes, i.e. *n* = 0. However, when the V_*MPE*_ is thermally diffuse in EC materials, according to equation (1), an EC enhancement function ${f}_{n}(t)=1+(n+1){t}^{n}$ and $t+(n+1){t}^{n+1}$ immediately operates on both the ∆*S* and ∆*T*. It should be noted that *C*_v_ is considered herein as a constant and that the influence of depolarization field and interfacial screening effect in the EC structures is not taken into account within equation (2).

Noting the irrelevance of *f*_*n*_(*t*) with *E* and the definition of ECE responsivity of ∆*T*/*E*, a function of *f*_*u*_/*u* can describe the *E*-dependence of ECE responsivity as well. We find that equation (2) limits the upper bound of EC entropy change for the EC materials with non-diffuse V_*MPE*_ as (−∆S)_max_ = ln(2)Ω*k*/V_*cr*_, which tallies with the (−∆S)_max_ result of thermodynamic and statistical mechanics^27^. Moreover, truncating the correlation length in nanocomposited or nanoconfined EC materials can enhance simultaneously the ∆*T* maxima and (−∆*S*)_max_, both of which are urgently required in realistic EC refrigeration applications^1, 18^.

The functional dependences of ${f}_{u}(E,t)$ and ECE responsivity on a normalized *E* are plotted in Fig. 1(b), where three distinct changing trends are underlined for ${f}_{u}(E,t)$ at a specific *T*: an initial quadratic (${f}_{u}={u}^{2}/2$) increase, a further linear $({f}_{u} \sim E)$ increment and gradual saturation at ultrahigh *E*. On the contrary, ECE responsivity is revealed as a non-monotonic function of *E*, but can still be approximated by a linear growth and then an exponential decay as *E* increases from 0. The acquired quadratic increase of EC entropy and temperature change with *E* successfully reproduces the ∆*T* changes with *E* ^2^ in dipole glasses^28^ measured as early as in 1965 and the recently-proposed quadratic scaling law of ∆*T* in PbZrO_3_ AFE^29^. It is also worth noting that both the proportional increase and the saturating trend of either ∆*T* or (−∆*S*) are frequently observed in pretty rich low-dimensional EC materials^30–34^.

Figure 1(c) illustrates the *n* and *E* influence on the thermal evolution of the enhancement function for ∆*T* and the *f*_*u*_(*E*, *t*) function. Taking an EC material with *n* = 10 for example, *f*_*u*_(*E*, *t*) exhibits abrupt change only over a narrow *T* interval and slight above T_*cr*_, which are also not very sensitive to *E* increase even by as much as a factor of 10. This is in sharp contrast to the *n* = 2 EC materials like PMN, where both *f*_*n*_(*t*) and *f*_*u*_(*E*, *t*) disperse over a wide *T* range between T_*cr*_ and 3T_*cr*_ and an amplified *E* effectively shifts a specific value of *f*_*u*_(*E*, *t*) towards higher temperatures. It can be seen that these observations capture the main features of 1^st^ order FE and 2^nd^ order relaxor phase transitions, and therefore ensure equation (2) is feasible for quantifying the EC cooling responses from adiabatic electric depolarization in sharp and diffuse phase transitions.

**Figure 2:**
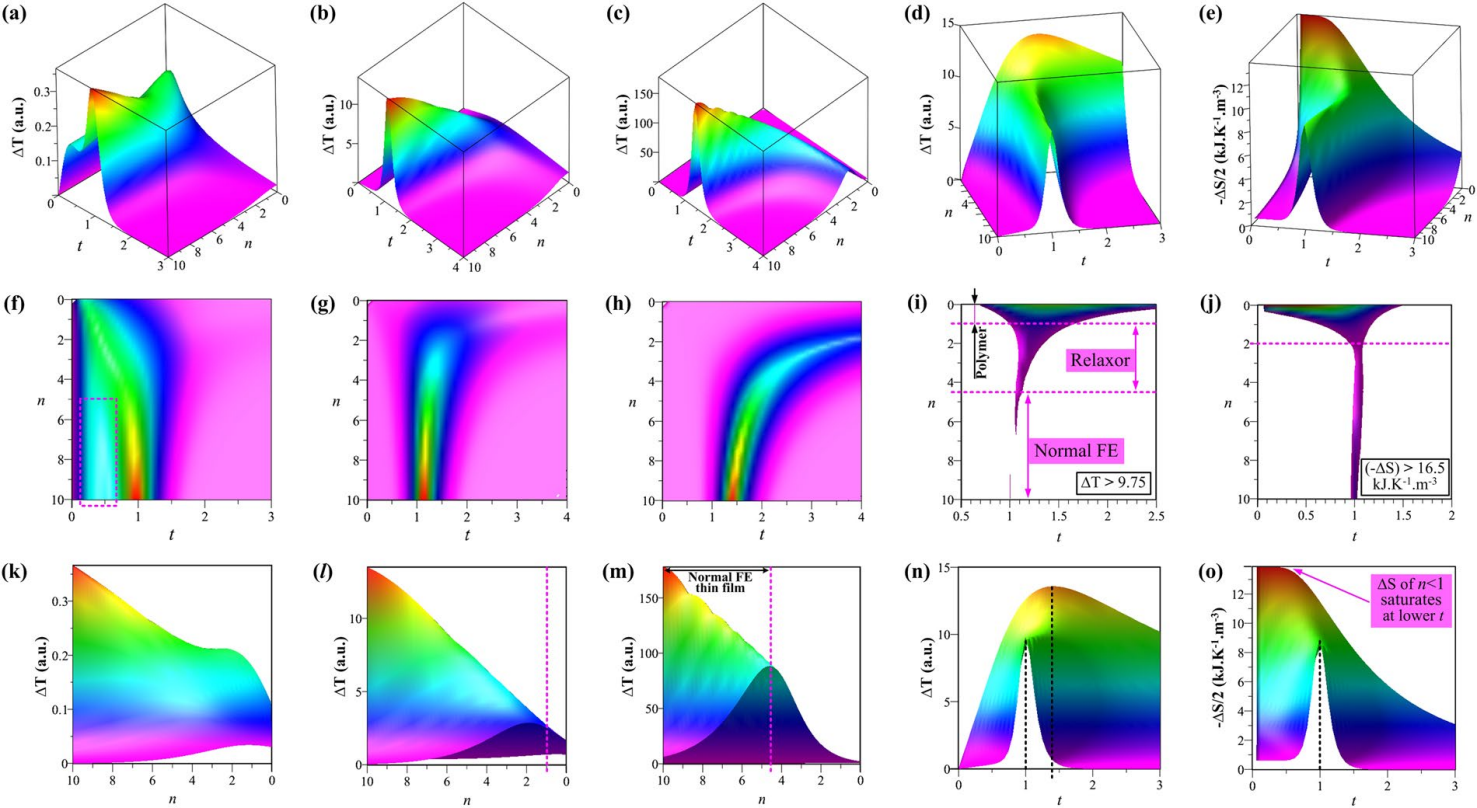
3D plots of our calculated EC cooling performances vs. *t* and *n*. Calculated ∆T surfaces of EC materials with a Small (**a,f,k**), Medium (**b,g,l**) and High (**c,h,m**) differentiation factor. The 3D surfaces of (**d,i,n**) ∆T and (**e,j,o**) (−∆S) are calculated for the medium factor case using V_*cr*_ = 7.28(1 + *n*). The panels in lower two rows are projections of the corresponding ones in the first row; (**i**) and (**j**) shows projected partial surface of ∆T ≥ 9.75 in (**d**) and (−∆S) ≥ 16.5 in (**e**) onto the *n* - *t* plane, respectively. (**k–m**) are attained by projecting (**a–c**) for *t* = 0.5–2. The dashed box in (**d**) marks the region of 2^nd^ peak in (**a**) at lower *T* than Curie temperature where the principle ∆T peak takes place.

We consider three categories of EC materials and low-dimensional structures based on a differentiation factor of *E*_*b*_ *T*_*cr*_ (herein *E*_*b*_ is dielectric breakdown field) - bulk FE, normal FE thin-films with long-range correlation and films of diffuse EC materials. These should possess a small, high and medium differentiation factors, respectively. Their three dimensional ∆*T* changes with *n* and *t* are calculated at first for a constant V_*cr*_-dominated coefficient in equation (2) and are illustrated in Fig. 2(a–c). As expected, ∆*T* in lower-*n* FE materials has an expanded peak across a broad *T* interval above *T*_*cr*_; whereas the ∆*T* in high-*n* EC materials maximize and decay steeply around *T*_*cr*_ and likely has a second peak corresponding to the so-called dual EC peaks phenomenon^35^ [Figures SI2(a-b)]. We particularly examine the case with a medium differentiation factor because it closely resembles typical currently-studied EC low-dimensional structures. An EC material with a lower *n* is shown to have ∆*T* maxima at higher *T* than that of higher-*n* FEs. Based on an absence of ∆*T* peaks observed in PVDF-based polymer FEs below the melting point^5, 15^ and commonly observed ∆*T* peaks in normal FE thin films^7, 11^, projected ∆*T* 3D surfaces are given in Fig. 2(l,m) by restricting *t* between 0.5*T*_*cr*_ and 2*T*_*cr*_ setting *n* < 1 for relaxor organic FEs and *n* > 4.5 for normal FE ultrathin films, with *n* = 1–4.5 for relaxor oxide EC materials. These *n*-assignments are marked in Fig. 2(i), which shows the projected ∆*T* surface of the medium factor EC materials in Fig. 2(d) that is computed by multiplying all the three functional terms in equation (2) and base on a proportionality between *n* and V_*cr*_. Accordingly, the overall (−∆*S*) surface and related projections under the same conditions are plotted in Fig. 2(e,j,o). It is apparent that both EC ∆*T* and ∆*S* peak at *T*_*cr*_ at the diffusionless phase transitions of normal FEs and that their maxima shift to higher temperatures by 0.4T_*cr*_ (i.e. over one hundred K since most EC materials have a T_*cr*_ above 300 K) as *n* decreases from 10 to 0 in diffusive depolarized EC materials.

Both the ∆*T* and ∆*S* display similar changing patterns for *n* = 2–10; however, their maxima gradually move to higher and lower temperatures (respectively) as *n* decrease from 2 to 0 [see Figures SI2(c-d)]. In particular, the *n* = 0 ∆*S* behavior is shown in Fig. 2(o) and SI3 and can be seen to monotonically increase with decreasing *t* and to saturate at *t* = 0. The overall picture shown by Fig. 2 and SI2 implies that, compared with normal FEs, ∆*T* and ∆*S* enhancements can be expected for *n* < 4.5 and *n* < 2 EC materials. This suggests organic FEs, relaxor EC materials and tuning strategies of reducing transition diffuseness should be considered in the race to accelerate the realization of flexible and room-temperature EC cooling technologies.

**Figure 3:**
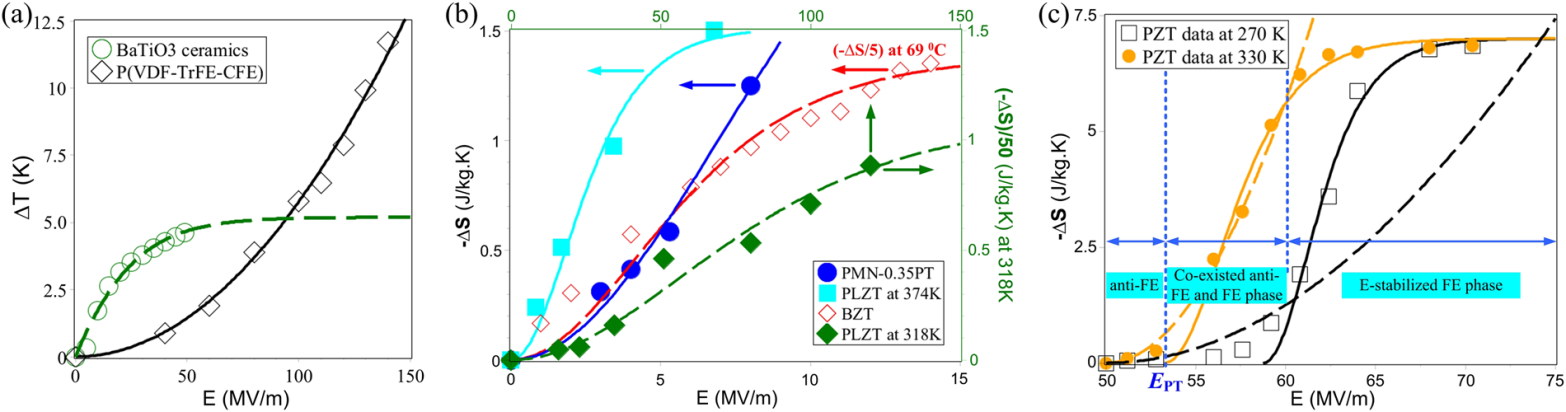
Theoretical predications and fittings for both ∆T and ∆S changes with *E* in representative EC materials. (**a**) Our fitting curves for experimental ∆T data in normal FE BaTiO_3_ (open circles) and relaxor P(VDF-TrFE-CFE) 59.2/33.6/7.2 mol% terpolymer (open boxes). (**b**) Fittings of the ∆S data in 0.65PMN-0.35PT, BZT and PLZT. (**c**) Direct comparison of calculated (−∆S) curves vs *E* with two sets of indirect experimental data in PZT AFE thin film. The BaTiO_3_, P(VDF-TrFE-CFE), 0.65PMN-0.35PT, BZT, PLZT and PZT data is derived from refs 11, 30, 31, 32, 33 and 34, respectively.

Our model has also been applied to available ∆*T* and ∆*S* data from literature to further verify its versatility. BaTiO_3_^11^ and P(VDF-TrFE-CFE) 59.2/33.6/7.2 mol% terpolymer^30^ film have been chosen as a representative normal FE and organic EC structures, respectively, and their directly-measured ∆*T* changes with *E* are best fitted in Fig. 3(a) adopting equation (2). Within our EC framework, the as-shown rapid ∆*T* saturation in BaTiO_3_ ceramics follows from its long-range correlation character and hence a sizable V_*cr*_ while the P(VDF-TrFE-CFE) polymer undergoes incomplete growth even at *E* = 150 MV/m, confirming its ultrahigh breakdown field and extremely small V_*cr*_. These inferences are consistent with our fitting result for V_*cr*_ of 86.5 nm^3^ for BaTiO_3_ ceramics and V_*cr*_ = 2.6 nm^3^ for relaxor P(VDF-TrFE-CFE) terpolymer. Moreover, equation (2) has been applied to the *E*-dependence of ∆*S* in relaxor 0.65PMN-0.35PbTiO_3_ (0.65PMN-0.35PT)^31^, Ba(Zr_0.15_Ti_0.75_)O_3_ (BZT)^32^ and (Pb_0.88_La_0.08_)(Zr_0.65_Ti_0.35_)O_3_ (PLZT)^33^ films, as shown in Fig. 3(b). It can be seen that our curves tally with the experimental data, except that the BZT curve underestimates the entropy change at lower *E*. This slight discrepancy in BZT is probably caused by the strong *E*-dependence of its correlation volume - although we determine V_*cr*_ = 71 nm^3^ for BZT film (comparable to that of BaTiO_3_ ceramics), there might also exist an *E*-induced AFE to FE phase transition in the BZT sample^32^. This suggests that such unique AFE to FE transitions need special consideration when calculating EC cooling properties of AFE materials, which is exemplified by Pb_0.95_Zr_0.05_TiO_3_ (PZT) thin films with its direct ∆*S* data at 270 K and 330 K, shown in Fig. 3(c). In particular, the stabilization of a long-range correlated FE phase is confirmed in the PZT film, formed out of an initial tiny AFE phase when *E* exceeds a threshold (*E*_TP_ = 53.3 and 58.8 MV/m at 330 K and 270 K). The PZT ∆*S* curve therefore constitutes three parts - a pure AFE region, a region of coexisting AFE and FE phases and an *E*-stabilized FE phase region. The V_*cr*_ is calculated as 29 nm^3^ and 29.8 nm^3^ for the FE phase at 330 K and 270 K, respectively, and as 3.2 nm^3^ and 1.3 nm^3^ for the AFE phase. The latter two are useful to explain the smaller *E*_TP_ at 330 K, because it is well-known that stabilizing a long-range order parameter in a matrix of larger polarizable elements requires less energetic activation. Our results indicate that the *E*-stabilized FE phase in PZT thin film is nearly *T*-independent and, via the V_*cr*_-dominated coefficient in equation (2), dominates the convergence of already saturated ∆*S* curves at ultrahigh *E*.

**Figure 4:**
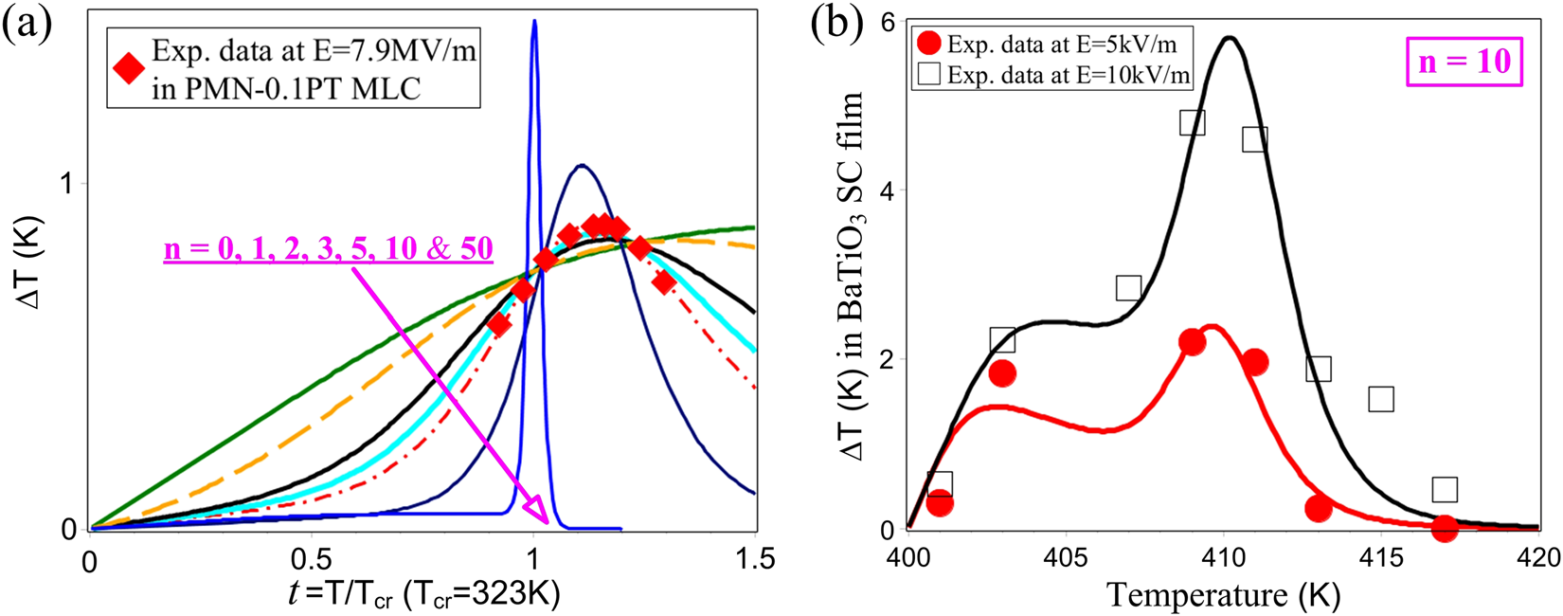
(**a**) Calculated ∆T curves based on equation (2) with various *n* and correspondence to directly measured ∆T data in 0.9PMN-0.1PT MLCs at *E* = 7.9 MV/m. (**b**) ∆T predications of equation (2) using *n* = 10 for two sets of directly measured ∆T data in BaTiO_3_ single crystal (SC) film subjected to *E* = 5 kV/m and 10 kV/m. The experimental data in (**a**) and (**b**) is derived from ref. 16 and ref. 11, respectively.

In addition to the proven versatility in quantifying the *E*-dependence of cooling responses in representative low-dimensional EC materials, equation (2) has been utilized to characterize EC responses as a function of temperature and contact with available data in literature. Directly measured ∆*T* data in normal FE BaTiO_3_ single crystal films^11^ and relaxor 0.9PMN-0.1PT multilayer capacitors (MLCs)^16^ illustrated in Fig. 4 serve as our database for our calculations of EC ∆*T* changes with *t*. The ∆*T* change in 0.9PMN-0.1PT MLCs shows a broad dispersion between 0.6 K and 0.9 K over a wide temperature range of 120 K; whereas, subjected to *E* = 10 kV/m, the BaTiO_3_ ∆*T* rises steeply upto 4.8 K and abruptly decays to zero within a rather small temperature range of less than 5% T_*c*_. These dissimilar thermal dispersions of ∆*T* can be considered as a natural consequence of distinct quasi-1^st^ order phase transition in normal FE BaTiO_3_ and the highly diffused depolarization dynamics in the relaxor 0.9PMN-0.1PT. Our ∆*T* calculations, on the basis of equation (2) with *n* = 0, 1, 2, 3, 5 and 10, are plotted in Fig. 4(a) for a direct comparison with the 0.9PMN-0.1PT data, which suggests that the *n* of 0.9PMN-0.1PT MLC lies between 2 to 5. The ∆*T* curve of an unrealistically high *n* = 50 is also plotted for comparison and may be regarded as the limiting case that recovers the EC features of a 1^st^ order transition. It can be seen that the ∆*T* vs. *t* curves, as a whole, reproduce the major figures of merits of distinctly diffused EC materials - a larger *n* essentially brings about a sharper but bigger EC peak close to *T*_*cr*_; diffusive phase transitions are manifested in a broad EC peak dispersion, yet lower in magnitude and located far above T_*cr*_. Our ∆*T* calculations yield V_*cr*_ = 21 nm^3^ for the 0.9PMN-0.1PT relaxor, which is appropriately larger than that in the PMN ceramics, V_*cr*_ = 16 nm^3^ determined from the ECE responsivity data^36^ in Figure SI(4), since the normal FE nature of PT solid is expected to increase the correlation length and strength of relaxor 0.9PMN-0.1PT solid solutions.

In order to avoid meaninglessly high values of *n*, our ∆*T* calculations for the BaTiO_3_ single crystal were restrained within a narrow *T* range of 400 K to 420 K where noticeable ∆*T* changes are observed. In view of the lower *E* (than the *E* for the 0.9PMN-0.1PT MLC) which was applied to adiabatically depolarize the BaTiO_3_ films, the novel implications of Figs 2(a) and SI2(a) predict the possible occurrence of the dual peak phenomenon in the measured BaTiO_3_ films. This can be used to explain the obvious shoulder in the ∆T change for BaTiO_3_ at *E* = 10 kV/m prior to the occurrence of the principle peak exactly at the Curie temperature and to clarify the absence of a clear maximum in ∆*T* at *E* = 5 kV/m. We choose the T_*cr*_ of BaTiO_3_ as 410 K and 410.5 K for the *E* = 5 kV/m and 10 kV/m data, respectively, while maintaining other parameters for the two ∆T datasets from BaTiO_3_. A value of V_*cr*_ = 108 nm^3^ was determined for the BaTiO_3_ single crystal film in Fig. 4(b), which is larger than the value of V_*cr*_ = 86.5 nm^3^ that we obtained for BaTiO_3_ ceramics films in Fig. 3(a). This expected V_*cr*_ decease in ceramic samples, along with significantly improved breakdown fields, underlines the feasibility of ceramic processes in reducing the correlation length and broadening the electric de-poling scope, thus offering promising routes to enhancing the cooling properties of strongly-correlated oxide EC materials.

## Discussion and Conclusions

We present a versatile EC theory, based on a combination of a Master equation and Maxwell relations, to analytically correlate the macroscopic EC cooling responses with sharp and diffusive phase transition characteristics. The adiabatic application of increased electric fields is found in equation (2) to trigger a quadratic increase of both ∆*T* and (−∆*S*). Subsequently, these quantities increase linearly with *E* and ultimately saturate at high enough *E*. It is worth noting that a steep exponent decline of the change in ∆*T* with *E* from 1 to 0.6 has been recently been reported near the morphotropic phase boundary in doped lead-free (Na_0.5_Ba_0.5_)TiO_3_-PT ceramic^9^ EC films. Closely examining equation (2) allows one to approximate the final saturation trend as $\sim (-2u/{e}^{2u})$, which further leads to an exponential decay of ECE responsivity as *E* increases. The latter, together with the initial quadratic and then linear increases of the ∆*T* change, gives an overall picture of the *E*-dependence of ECE responsivity, which may be used to better understand the ECE responsivity data found in literature [see Figure SI(4)]. Also, the proportionality of the ECE responsivity maxima to *P*_*max*_ requires piecewise functions for AFEs such that the *E*-induced AFE to FE phase transition and resultant *P*_*max*_ increase be included to represent the typical double *P*-*E* hysteresis loops^4^ in AFE EC materials.

**Figure 5:**
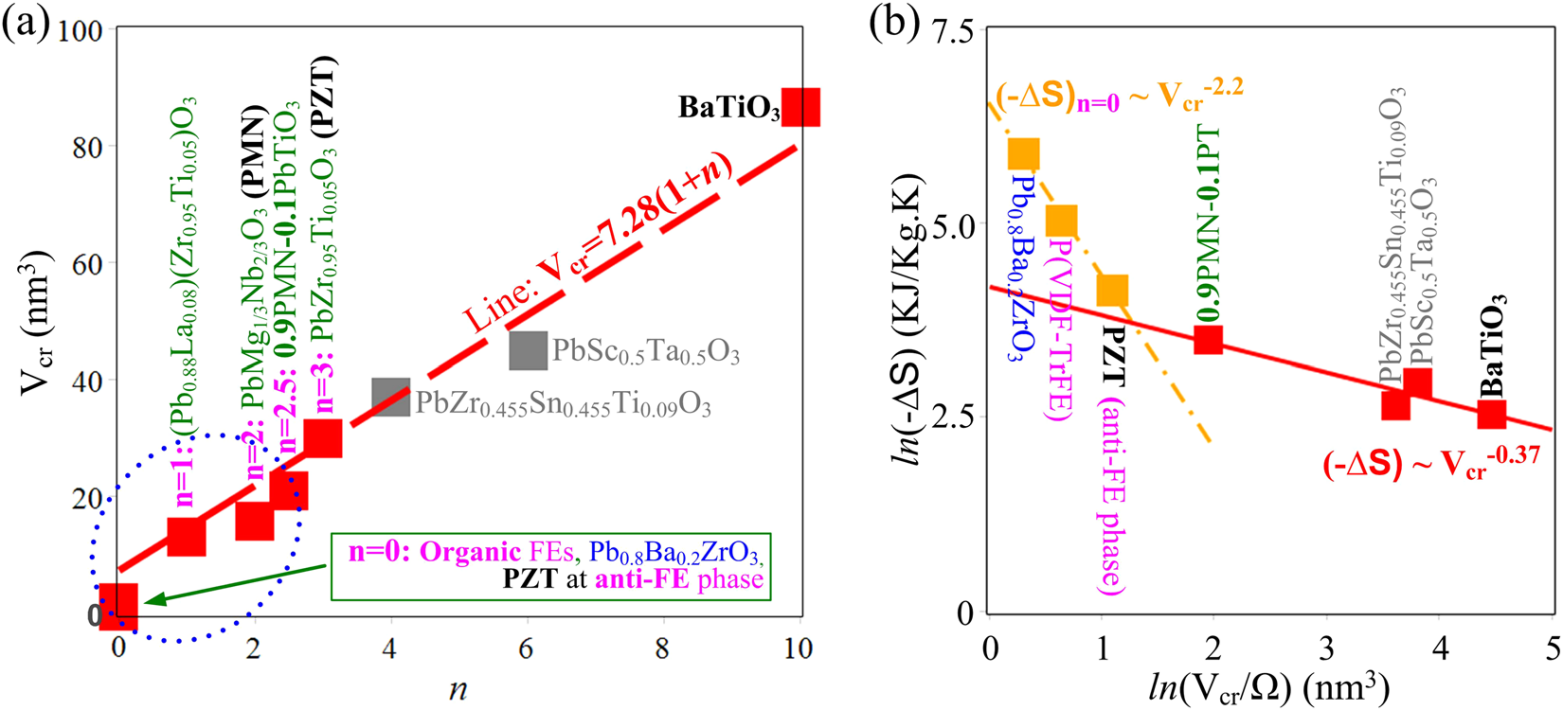
A linear dependence of correlation volume on transition diffuseness index and Distinct scaling laws of ∆S in typical EC materials. (**a**) A linear relation between V_*cr*_ and *n* in typical EC materials. (**b**) Distinct scaling laws of ∆S in *n* > 2 EC materials exemplified by BaTiO_3_ normal FE and relaxor FEs and in *n* = 0 EC materials including Pb_0.8_Ba_0.2_ZrO_3_ oxide, AFE Pb_0.95_Zr_0.05_TiO_3_ and FE P(VDF-TrFE) 65/35 mol.% copolymer. The symmetry factor Ω is 3 for 0.9PMN-0.1PT and 1 for others. The dotted ellipse encloses the EC materials expected to possess significantly enhanced entropy change. The ∆S data in (**b**) is derived from ref. 1.

Based on the correlation volume results obtained from our theoretical fittings of equation (2) for the adopted EC responsive data in Figs 3, 4 and SI4 for a rich range of representative EC low dimensional structures and then V_*cr*_ calculations based on the derived $u(E,T)$ equation, we are able to determine the transition diffuseness index of these studied EC materials and our *n* results are shown in Fig. 5(a). Universal linearity between V_*cr*_ and *n* is evident. It is striking yet critical to develop this generic linearity since it could directly explain the divergent sharpness and diffuseness of observed EC responses to localized correlation volume and activation energies for adiabatic depolarizations. It is also worth noting that such a functional bridge can span over substantial length scales, exemplified by the calculated values of V_*cr*_ = 1.9 nm^3^ and 1.6 nm^3^ for P(VDF-TrFE) copolymer and Ba(Zr_0.15_Ti_0.75_)O_3_, to as high as ~100 nm^3^ for BaTiO_3_. In view of the fact that the BaTiO_3_ V_*cr*_ acts merely at its FE domain walls, which separate FE domains with much longer dimensions and effectively mediate *E*-induced polarization switching, it is therefore useful to postulate that the *n*-V_*cr*_ linear correlation bridges EC studies from micrometer domain transition scale down to unit cell repeat scales. However, within such a large spread across such broad length scales, the dimensionality of EC phase transitions and the surrounding elastic and electronic conditions are not expected to be identical, and this in turn significantly alters the EC cooling behaviors.

The effects of EC transition dimensionality on ∆*S* scaling behaviors are distinguished in Fig. 5(b). It can be seen ∆*S* in *n* = 0 EC materials, including polymeric FEs, AFE phase Pb_0.95_Zr_0.05_TiO_3_ and Pb_0.8_Ba_0.2_ZrO_3_, scales proportionally with V_*cr*_^−2.2^; whereas ∆*S* in Pb_0.95_Zr_0.05_TiO_3_, relaxor and normal FE EC materials scales distinctively as ∆S ~ V_*cr*_^−0.37^. Recalling the extremely small (<3 nm^3^) correlation volume in the *n* = 0 EC materials and the recently-proposed inversely quadratic relation of ∆*S* with correlation length^37^, ∆S ~ V_*cr*_^−2.2^ is fundamentally ascribed to one-dimensional structural confinements of lattice-scale depolarization dynamics in the *n* = 0 EC materials and structures and as well as in the remaining two degrees of freedom of concomitant entropy changes. These novel findings suggest that the upper bound of EC entropy change can be limited by the unit cell volume. Thus replacing the V_*cr*_ in our derived (−∆*S*)_max_ = ln(2)Ω*k*/V_*cr*_ with unit cell volumes reported in ref. 27 permits us to predicate values for (−∆*S*)_max_ as high as 300 kJ/(K.m^3^) and 215 kJ/(K.m^3^) for Pb_0.8_Ba_0.2_ZrO_3_ and P(VDF_0.65_TrFE_0.35_), respectively. In contrast, the distinct scaling of ∆*S*_max_ ~ V_*cr*_^−0.37^ in rich relaxor and normal FE EC materials tallies with the theoretically demonstrated long-range dipolar correlation exponent of 2/3 in rough polar interfaces^38, 39^. The well-known FE domain wall dimensionality of 2.5 and fractional dimensionality of PNR evolutional dynamics in relaxors^21, 26^ are also essentially responsible for the ∆*S*_max_ ~ V_*cr*_^−0.37^ scaling.

Our findings provide guidance for characterizing known low-dimensional EC materials and designing new ones with enhanced cooling performance by deliberately increasing phase transition diffusivity and shortening the correlation length. Following this logic, the already promising EC responses found in the *n* = 0 EC materials and low-dimensional structures, which can be exemplified by polymeric FEs and AFEs as well as their phase-coexisting nanocomposites with the minor phase of nanostructured normal FEs (like nanowires^40^ and nanoparticles^15^), together with the illustrated much higher sensitivity of their EC enhancements to decreased characteristic correlation volume by introducing chemical doping, structural nanoconfinement and truncation, highlights the unparalleled potential of these phase-coexisting *n* = 0 EC low-dimensional nanostructures and (polymeric or doped) nanocomposites as the ultimate solution to materialize the long-sought all-solid-state refrigeration and on-chip thermal management. Based on this V_*cr*_-reducing or phase-coexisting material design strategy, it is important to point out that some new types of prospective flexible *n* = 0 EC materials can be expected via either compositional engineering or compositing polymeric FE matrix and nano-fillers of AFEs or phase-coexisting FEs. The distinct scaling laws of maximum EC entropy change have potential applications in detecting new minority phases in phase-coexisting EC materials and for extrapolating electric and thermal activations for which current EC cooling data are not available yet. Also, our work may have critical implications beyond EC cooling properties, as an analogous theoretical framework could be established for other extensively-studied caloric effects such as magnetocalolric and mechanocaloric effects.

## Methods

The Master equations are introduced to derive a generic expression for the macroscopic polarization in EC materials as a function of thermal and electric activations. The defined microscopic polar elements (MPEs) are assumed to be embedded within a paraelectric matrix, but mutually correlated at a variety of length scales. At sufficiently high *T* and zero *E*, the localized dipoles in EC MPEs are randomly oriented, cancelling out the overall polarization for ECE. When activated by thermal cooling or *E*-poling along the axis of lattice symmetry, the polarization of partial MPEs reorientates and aligns along the closest lattice-symmetry allowed direction, leading to the emergence and increase of overall polarization magnitude and hence the EC entropy decrease. The thermal cooling and *E*-activated partial alignments of localized polarization among all the MPEs lead to an equivalent number density of MPEs occupying a favored ground state. The time (*t*)-dependent probability (*p*_*n*_) of the *n* ^th^ ground state occupied by the EC MPEs is governed by the Master equations:

3 $$\tau \frac{d}{dt}{p}_{n}(t)=\sum _{m\ne n}[{p}_{m}(t)-{e}^{\mu (n,m)}{p}_{n}(t)]$$

where integer *m* ≠ *n*, *τ* denotes characteristic relaxation time and *µ*(*n* ↔ *m*) is the energy difference between the *m* ^th^ and *n* ^th^ state. For an EC material with two ground states, *µ*(0,1) = *Q*V_*MPE*_/*kT* with *k* Boltzmann’s constant. Here *Q* corresponds to the activation energy density given by 2*E* × *P*_*max*_ with *P*_*max*_ denoting an *E*- and *T*-independent polarization maxima attainable in the two-state system. Although the frequency-dependent depolarization dynamics and the energy contribution of varied density of domain walls or interfaces in polarizable materials to our defined *Q* as well as their effects on the overall EC response could be further considered within this proposed framework by including both interfacial energies^41^ and time-related solutions to the formulated Master equations, these factors are not particularly formulated due to their inessential roles and associated complexity and, thus, call for our future studies.

At equilibrium (in the limit of time *t* → ∞) and under *E*-poling applied along the axis of lattice symmetry, the Master equations give a generic expression of the macroscopic polarization magnitude (*P*):

4 $$P={P}_{\max }\,\tanh (E{P}_{\max }{{\rm{V}}}_{MPE}/{\rm{\Omega }}kT)$$

where $u(E,T)=E{P}_{\max }{{\rm{V}}}_{MPE}/({\rm{\Omega }}kT)$ represents a universal activation parameter of both *E* and *T*; Ω is a symmetry contribution factor equaling to 1 and 3 for a two-state (e.g. tetragonal and polymeric) and eight-state (e.g. rhombohedral) EC material, respectively.

## Electronic supplementary material

Supplementary Information
