# Supplementary material for: A scaling law for distinct electrocaloric cooling performance in low-dimensional organic, relaxor and anti-ferroelectrics
Source: Sci Rep. 2017 Sep 11;7:11111. doi: 10.1038/s41598-017-11633-y (PMC5593997; doi:10.1038/s41598-017-11633-y)
Supplement: Supplementary file 1 — Supplementary Information [file 41598_2017_11633_MOESM1_ESM.pdf]

Electronic Supplementary Information (ESI) to:

A scaling law for distinct electrocaloric cooling performance in low-dimensional organic, relaxor and anti-ferroelectrics

Yuping Shi[1,2,3], Limin Huang[2,*], Ai Kah Soh[3], George J. Weng[4], Shuangyi Liu[5] & Simon A.T. Redfern[6,7]

[1]Department of Mechanical and Aerospace Engineering, Hong Kong University of Science and Technology, Clear Water Bay, Kowloon, Hong Kong.

[2]Department of Chemistry, South University of Science and Technology of China, Shenzhen 518055, China.

[3]School of Engineering, Monash University Malaysia, Bandar Sunway 46150, Malaysia.

[4]Department of Mechanical and Aerospace Engineering, Rutgers University, New Brunswick, New Jersey 08903, USA.

[5]Chongqing Institute of Green & Intelligent Technology, Chinese Academy of Sciences, Chongqing 400714, China.

[6]Department of Earth Sciences, University of Cambridge, Downing Street, Cambridge CB2 3EQ, UK.

[7]HPSTAR, 1690 Cailun Rd, Pudong District, Shanghai 201203, China.

[*]Correspondence and requests for materials should be addressed to L.H. (email: huanglm@sustc.edu.cn)

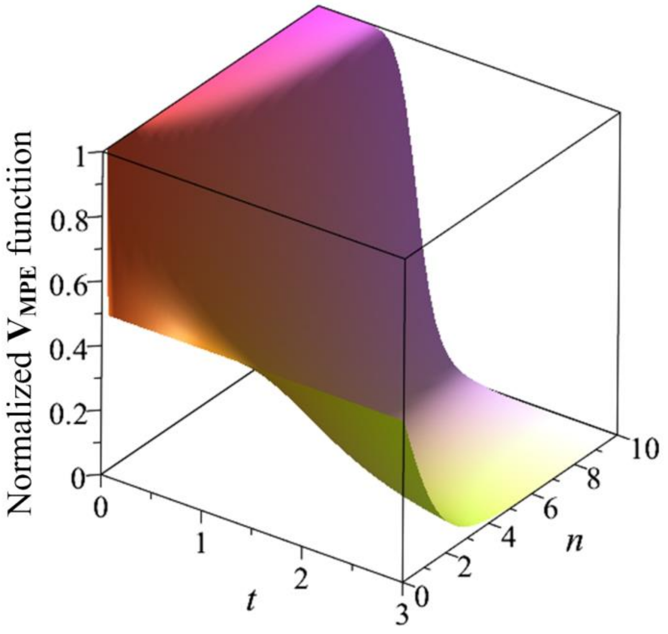

Figure S11. A 3D plot of differently diffused MPE volume (normalized by V_{cr}) in EC materials as a function both *t* and *n*.

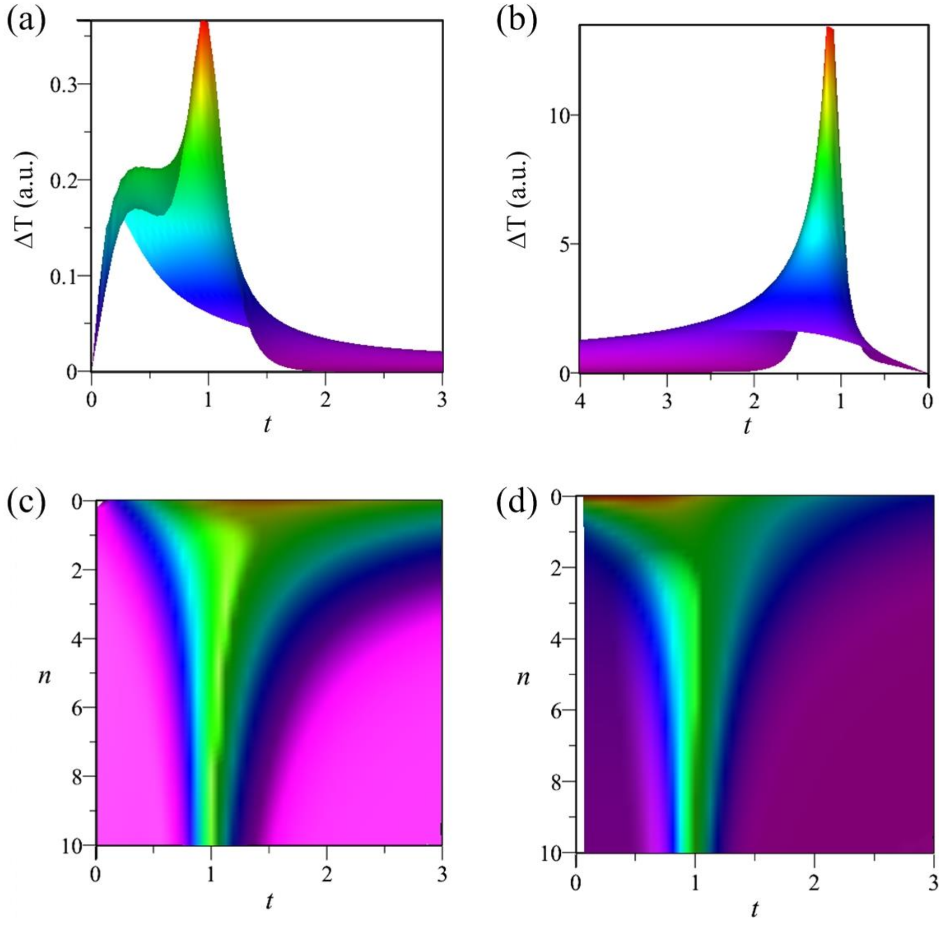

Figure SI2. (a, b) ΔT-*t* plane projection of calculated ΔT 3D surfaces in Figures 2(a) and 2(b) in the main paper; (c, d) *n*-*t* plane projection of the ΔT and (-ΔS) 3D surfaces shown in Figures 2(d) and 2(e).

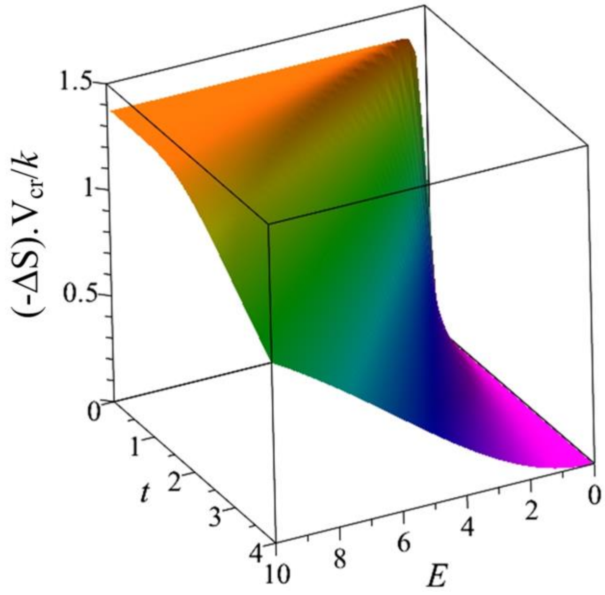

Figure SI3. A 3D plot of negative entropy change (normalized by k/V_{cr}) as a function of both *t* and *E* activations in the *n*=0 electrocaloric materials and structures.

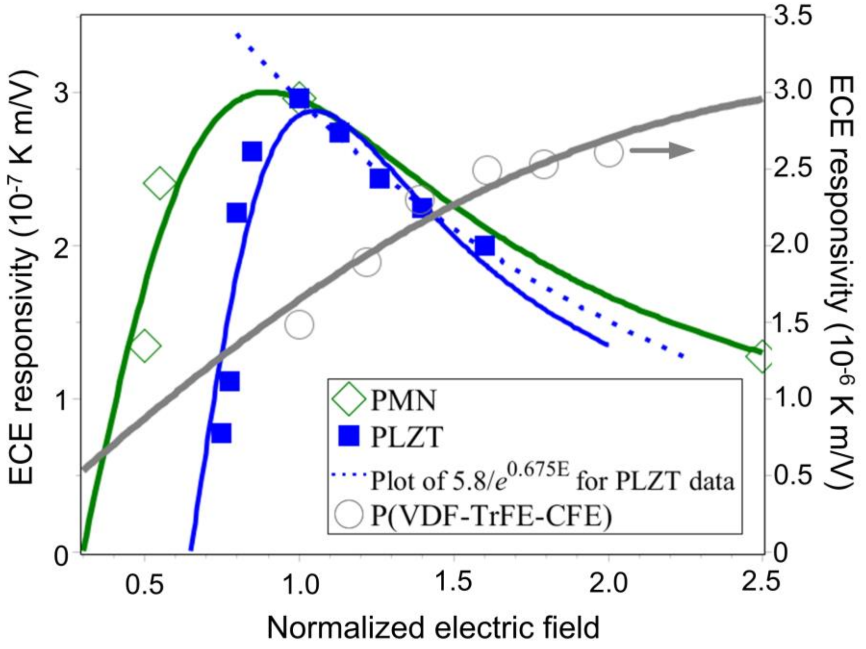

Figure SI4. Our ECE responsivity fitting curves for the experimental data measured in PMN, PLZT and P(VDF-TrFE-CFE). An exponential decay is also plotted for the PLZT data for a direct comparison. The PMN and PLZT data are derived from ref. 36; P(VDF-TrFE-CFE) data are from ref. 10. Reference codes and material abbreviations refer to the main paper.
